# SOCIAL PARTICIPATION AND DEPRESSIVE SYMPTOMS AMONG SPOUSAL AND ADULT CHILD CAREGIVERS

**DOI:** 10.1093/geroni/igac059.984

**Published:** 2022-12-20

**Authors:** Elliane Irani, Fei Wang

**Affiliations:** Case Western Reserve University, Cleveland, Ohio, United States; Case Western Reserve University, Cleveland, Ohio, United States

## Abstract

Participating in social activities through formal (e.g., social or religious organizations) and informal (e.g., gatherings with family members or friends) avenues is known to positively contribute to mental wellbeing. Family caregivers are at risk for limited social participation and increased depressive symptoms. However, little is known about which social activities are associated with depressive symptoms among spousal and adult child caregivers. The purpose of this study was to examine the effects of participating in various social activities on depressive symptoms among spousal (n=422) and adult child (n=1,112) caregivers using data from the 2017 wave of the National Study of Caregiving. Caregivers reported on participating in six social activities in the past month (e.g., visiting family and friends, attending religious services, doing volunteer work, working for pay) and completed the 2-item Patient Health Questionnaire to assess depressive symptoms. Data were analyzed using multiple linear regression, with separated models for spousal and adult child caregivers. Sociodemographic characteristics, self-rated health, and dementia caregiving status were included as covariates. For spousal caregivers, visiting friends or family and attending religious services were associated with lower depressive symptoms (b=-.55, p=.010 and b=-.33, p=.036, respectively). For adult child caregivers, going out for enjoyment was associated with lower depressive symptoms (b=-.80, p<.001). Findings suggest that spousal and adult child caregivers may benefit from participating in different types of social activities. Interventions targeting social participation to reduce depressive symptoms need to be tailored to the needs and preferences of spousal and adult child caregivers to be most effective.

